# *Athrixia phylicoides* tea infusion (bushman tea) improves adipokine balance, glucose homeostasis and lipid parameters in a diet-induced metabolic syndrome rat model

**DOI:** 10.1186/s12906-021-03459-z

**Published:** 2021-11-29

**Authors:** Madigoahle A. M. Mokwena, Godwill Azeh Engwa, Benedicta N. Nkeh-Chungag, Constance R. Sewani-Rusike

**Affiliations:** 1 Department of Human Biology, Faculty of Health Sciences, Walter Sisulu University PBX1, Mthatha, 5117 South Africa; 2 Department of Biological and Environmental Sciences, Faculty of Natural Sciences, Walter Sisulu University PBX1, Mthatha, 5117 South Africa

**Keywords:** *Athrixia phylicoides*, High-energy-diet, Insulin resistance, Obesity, inflammation, Metabolic syndrome

## Abstract

**Background:**

Central obesity and insulin resistance are associated with metabolic syndrome (MetS) which is aggravated by diet and sedentary lifestyle. *Athrixia phylicoides* (AP) is reported by rural communities to have medicinal benefits associated with MetS such as obesity and type 2 diabetes. This study was aimed to investigate the effects of AP on diet-induced MetS in Wistar rats to validate its ethnopharmacological use.

**Methods:**

AP was profiled for phytochemicals by LC-MS. After induction of MetS with high energy diet (HED), 30 male rats were divided into five treatment groups (*n* = 6): normal diet control, HED control, HED + AP 50 mg/Kg BW, HED + AP 100 mg/Kg BW and HED + 50 mg/Kg BW metformin. The rats were treated daily for 8 weeks orally after which weight gain, visceral fat, total cholesterol, free fatty acids (FFAs) and adipokine regulation; leptin: adiponectin ratio (LAR) were assessed. Also, glucose homeostatic parameters including fasting blood glucose (FBG), oral glucose tolerance test (OGTT), glucose transporter 4 (GLUT 4), insulin and homeostatic model assessment of insulin resistance (HOMA-IR) were determined.

**Results:**

Findings showed that AP was rich in polyphenols. The HED control group showed derangements of the selected blood parameters of MetS. AP reversed diet-induced weight gain by reducing visceral fat, total blood cholesterol and circulating FFAs (*p* ≤ 0.05). Treatment with AP improved adipokine regulation depicted by reduced LAR (*p*<0.05). Treatment with AP improved parameters of glucose homeostasis as demonstrated by reduced FBG and HOMA-IR (*p* ≤ 0.05) and increased GLUT 4 (*p*<0.05).

**Conclusion:**

*Athrixia phylicoides* tea infusion was shown to possess anti-obesity and anti-inflammatory properties, improved glucose uptake and reduce insulin resistance in diet-induced MetS in rats which could be attributed to its richness in polyphenols. Therefore, AP could have potential benefits against type 2 diabetes and obesity which are components of MetS validating its ethnopharmacological use.

## Background

Obesity, dyslipidaemia and insulin resistance (IR) are considered key to the global rise in metabolic diseases including diabetes and cardiovascular diseases [1]. Obesity, especially central obesity, is considered to be the primary risk factor for the development of metabolic syndrome (MetS) disorders [2]. The metabolic factors include obesity, hypertriglyceridaemia, Low high density lipoprotein cholesterol (HDL-c), high blood pressure and high fasting blood glucose. The concurrence of at least three of these five risk factors defines a state of MetS [1]. The consumption of high fat/energy diet (HED) and/or lack of physical activity leads to the accumulation of body fat due to sustained positive energy balance [3]. This fat is stored in adipocytes as triglyceride (TG) under the control of insulin. In obesity, excess visceral fat stored in the adipose tissue leads to hypertrophy of adipocytes and hypoxia which is associated with reduced insulin sensitivity accompanied by enhanced release of free fatty acid (FFA) into circulation [4]. The increased plasma FFA fluxes into the liver leading to hepatic accumulation of TG which may be converted to cholesterol [4]. Thus, increased hepatic TG (hypertriglyceridaemia) is the hallmark of dyslipidaemia in obesity as it leads to delayed clearance of TG-rich lipoproteins thereby promoting the formation of low density lipoprotein cholesterol (LDL-c) and decreasing HDL-c [5, 6]. Also, increased FFA is known to be negatively associated with HDL-c [7]. Elevated FFA release is known to inhibit insulin’s lipogenic action through the inhibition of lipoprotein lipase thereby increasing FFA release into the circulation. More so, increase in circulating FFAs is associated with decreased whole body insulin-stimulated glucose uptake thereby leading to IR [5].

Insulin resistance may also originate from obesity-induced inflammation [8]. Excessive growth of visceral adipose depots, associated with adipocyte hypertrophy results in the activation of inflammatory pathways. Obesity is involved in the alteration of adipokines; adiponectin and leptin which are known to regulate blood pressure, lipid and glucose metabolism and inflammation [9] and also associated with MetS [10, 11]. Leptin upregulates pro-inflammatory cytokines such as tumour necrosis factor-alpha (TNF-α) and interleukin (IL) -6 which are associated with IR [12]. In contrast, adiponectin which is an anti-inflammatory molecule downregulates the expression and release of many pro-inflammatory mediators [13], promotes vasodilation, regulates glucose and lipid metabolism and also improves insulin sensitivity [14, 15]. As such, increased leptin to adiponectin ratio (LAR) has been reported to be a good marker for assessing obesity-induced low-grade inflammation than isolated leptin or isolated adiponectin concentrations [16, 17] and has been shown to be strongly associated with MetS [16]. López-Jaramillo et al. further suggested that the LAR may be a useful diagnostic index for IR and a good marker for assessing the effectiveness of antidiabetic therapy [16]. Obesity-induced low-grade inflammation decreases insulin signaling which results in IR in liver and skeletal muscles [18]. IR is a metabolic state characterised by hyperinsulinaemia and hyperglycaemia in which cells fail to sufficiently respond to insulin action [19]. This impaired insulin response results in decreased uptake of glucose into cells as a result of impaired expression and translocation of glucose transporter 4 (GLUT4) in the muscle and adipose tissues [20].

Globally, there is increased interest in the use of medicinal plants in the management of MetS-related diseases as prescribed by community traditional healers and elders [21]. Traditional herbs rich in polyphenols have been shown to possess beneficial biological effects on components of the MetS [22]. Some of these herbs are easily accessible in certain local communities and often consumed as natural teas. *Athrixia phylicoides* (Bushman tea) is a plant with a notable history of its use as a tea by the indigenous people of South Africa and for the treatment of diabetes and hypertension [23]. This plant is rich in flavonoids and polyphenols with several medicinal properties including antifungal, antibacterial, antimalarial, anti-inflammatory and antioxidant activities [24, 25]. In vitro studies of *Athrixia phylicoides* (AP) extracts demonstrated increase glucose uptake and metabolism in myoblasts and adipose tissue cell lines [26] suggesting antidiabetic effects. However, there are no reports on the in vivo effects of AP tea infusion against MetS using animal model. This study therefore aimed to assess the effects of AP tea infusion on selected parameters of MetS in HED-induced rats.

## Materials and methods

This study protocol complied with relevant institutional, national, and international guidelines, regulations and legislation required for plant and animal studies.

### Source of plant material

*Athrixia phylicoides* was harvested from Lusikisiki, Eastern Cape Province of South Africa from its natural habitat and supplied by Mr. Fikile Mahlakata, a traditional healer. The plant was authenticated by Dr. Immelman, Department of Botany, Walter Sisulu University. Voucher specimen (Maganga 1 KEI) was archived in the Kei Herbarium of Walter Sisulu University (WSU).

### Phytochemical profiling by Liquid Chromatography- Mass Spectrometer (LC-MS)

Leaves of AP were dried at room temperature and manually crushed into a powder. The dried leave powder (2 g) was soaked overnight with 15 mL of 50% methanol in deionised water containing 1% formic acid and extracted for 60 min at room temperature in an ultrasonic bath (0.5 Hz, Integral Systems, RSA). Prior to LC-MS analysis, the extract was centrifuged (Hermle Z160m,) at 3000 g for 5 min. An ultra-performance liquid chromatography (UPLC) (Waters, Milford, MA, USA) connected to a Waters Synapt G2 Quadrupole time-of-flight (QTOF) mass spectrometer (MS) was used for LC-MS analysis as previously reported by us [27]. Calibration and calculations were done using polyalanine according to Rautenbach et al. [28]. Samples were run in triplicate and results reported as relative abundance (%).

### Preparation of tea infusion

Five grams (5 g) of AP dried leaves was steeped in 100 mL of boiling water overnight in a shaking incubator (Labcon 5082 U) at 60 °C. The following day, the tea was vacuum filtered using a Buchner funnel and Whatman™ No.1 filter paper. Tea infusion was made fresh every week for the duration of the treatment period. The tea has been reported to have no toxic effects even at high doses [29].

### Ethical considerations

All experimental procedures conducted in the study complied with the accepted ethical guidelines of Animal Care and Use and the Animal Research: Reporting of In Vivo Experiments (ARRIVE) guidelines and with the principles for laboratory animal use and care as specified by the South African National Standard (SANS 10386:2008) [30]. The study was approved by the Faculty of Health Science Research Ethics Committee, WSU with Protocol Number: 108/2018.

### Animal care

Thirty 12-week old adult male Wistar rats, weighing 280–350 g were purchased from the South African Vaccine Producers (Johannesburg, South Africa). The rats were housed three per standard polypropylene cage (460 × 310 × 140 mm) with pine shavings bedding in a temperature-controlled environment of 24–25 °C, with 12-h dark: light cycle. The animals were acclimatized for a one-week period to adapt to their new environment prior to commencement of the experiment. During the experimental period, animals were allowed free access to water, standard rat chow (EPOL, South Africa) and high energy diet (HED). HED was prepared as previously reported [31] with some modifications where sucrose was replaced with fructose as fructose has been reported to have greater effect than sucrose in inducing MetS [32]. The nutrient content of the standard rat chow and HED are summarized in Table 1.

**Table 1:** Feed composition and caloric value of normal and high energy diets

Normal Diet (ND)				Highly Energy Diet (HED)		
Ingredient	quantity (g/Kg)	% energy	caloric value (Kcal/Kg)	quantity (g/Kg)	% energy	caloric value (Kcal/Kg)
**Protein**	180	43.9	720	121.91	16.7	487.64
**Fat**	25	6.1	225	116.8	16	1051.2
**Carbohydrate**	60	14.6	240	491.29	67.3	1965.16
**Others**	145	35.4	–	–	–	–
**Total**	410	100	1185	730	100	3504

Others denotes: *vitamins, minerals (calcium, phosphorus, iron, trace elements) fibre, moisture and ash*

*g/Kg* grams per kilogram, *Kcal/Kg* kilocalorie per kilogram, *%* percent

### Study design

The induction of MetS in rat was done using a previous research protocol [33]. Animals were fed ad libitum with HED for 12 weeks and were assessed for IR after the intervention. After confirmation of IR via intraperitoneal insulin tolerance test (data not shown), Wistar rats were randomly allocated to groups (*n* = 6/group) and administered one of the following treatment regimens:

- Normal diet control (NDc) = Normal diet + distilled water.
- HED control (HEDc) = HED + distilled water.
- HED low dose (HED_L_) = HED + AP 50 mg/Kg body weight (BW),
- HED high (HED_H_) = HED + AP 100 mg/Kg BW.
- HED metformin (HED_M_) = HED + 50 mg/Kg BW metformin.

Tea infusion of 50 mg/Kg BW (2.5 ml/Kg BW) AP and 100 mg/kg BW (5 mL/Kg BW) AP is equivalent to 1 cup and 2 cups human dose respectively, using the body surface area as a conversion factor of daily therapeutic dose from human to rat using a multiplication factor of 6.2 according to Reagan-Shaw et al. [34]. These doses were comparable to that of metformin used in the study. Rats were treated for 10 weeks of experimental period, between 08:00–09:00 am daily by oral gavage. The rats were weighed every 2 weeks on an electronic balance (KERN_PLS_) to adjust AP dose relative to body weight over the 10-week treatment period. The amount of calories consumed and weight of the animals was measured to determine the net weight gain.

#### Calculations

Caloric intake (CI) per 100 g BW for ND and HED fed rats and treatment groups was calculated from feed consumed as:

$$CI/100g. BW=\frac{\left( total\ energy\ from\ diet\kern0.5em \left(\frac{Kcal}{Kg}\right)\times \frac{ total\ feed\ consumed\ \left(\frac{g}{Kg}\right)}{total\ weight\ of\ feed\ \left(\frac{g}{Kg}\right)}\right)100g}{total\ BW(g)}$$

Net body weight gain was calculated as:

$$final\ body\ weight\ (g)- initial\ body\ weight\ (g)= net\ body\ weight\ gain\ (g)$$

### Fasting blood glucose

To assess fasting glucose levels as a measure of glucose homeostasis, animals were fasted overnight for 8–12 h on the day of terminal procedures. Tail tips were pricked using a sterile lancet and blood glucose concentration (mmol/L) was measured using a calibrated Accu-Chek® glucometer (Roche Diabetes Care, Inc., USA).

### Oral glucose tolerance test

Oral glucose tolerance test (OGTT) was performed at week 8 of the treatment period to determine glucose clearance as a measure of endogenous insulin response in rats after a glucose challenge. The protocol was performed as previously described from our laboratory [35]. Briefly, animals were fasted overnight for 8–12 h and fasting blood glucose (FBG) was measured at time 0, using Accu-Chek® glucometer. The animals were given an oral glucose load of 2 g/kg BW through oral gavage. FBG levels in mmol/L were determined at 30, 60, 90 and 120 min. The data was used to construct a dose response curve (blood glucose vs time) and area under the curve was calculated using GraphPadPrism version 5® software (GraphPad Software Inc., San Diego, CA, USA) which employs the trapezoid method. Results were compared between the groups.

### Terminal procedures

After 10 weeks of treatment, final body weight and fasting glucose concentration were measured. Total blood cholesterol (mmol/L) was determined using EasyTouch® cholesterol meter (Sterilance Medical (Suzhou) Inc.) according to manufacturer’s protocol. Animals were terminated by CO_2_ inhalation in a closed container. Blood was collected by cardiac puncture using a 21G needle attached to 5 mL syringe into ethylenediaminetetraacetic acid (EDTA) vacutainer tubes for plasma collection. The blood was centrifuged for 15 min at 3000 rpm (Eppendorf 5810R) to obtain plasma which was aliquoted into Eppendorf tubes for each rat and frozen at − 20 °C for biochemical analyses. Left gastrocnemius muscle of each rat was collected and immediately frozen for the determination of glucose transporter 4 (GLUT 4) concentration. Visceral fat from the abdomen and epididymis fat pads was isolated and weighed for calculation of percent visceral fat (adipose tissue) accumulation as: $Adipose\ tissue\ \left(\%\right)=\left[\frac{visceral\ fat\ weight\ (g)}{fasting\ body\ weight\ (g)}\right]\times 100.$

### Biochemical analyses

Plasma leptin and insulin levels were assayed using enzyme linked immunosorbent assay (ELISA) kits (Elabscience catalogue: E-EL-R0582 and E-EL-R2466 respectively) while FFA and adiponectin were assayed using Abebio and Quantikine ELISA kits (catalogue AE43768RA and RRP300 respectively) as per manufacturer’s protocol.

### Calculation of homeostatic model assessment of insulin resistance

Fasting glucose concentration and plasma insulin concentrations were computed in the HOMA2 calculator v2.2.3 (https://www.dtu.ox.ac.uk/homacalculator/download.php) to determine homeostasis model assessment of insulin resistance (HOMAIR). Insulin resistance was calculated as $\mathrm{HOMA}-\mathrm{IR}=\frac{insulin\ \left(\mathrm{pMol}\right)\times fasting\ glucose\ \left(\frac{mg}{dL}\right)}{405}$

### Determination of gastrocnemius muscle glucose transporter 4

Gastrocnemius muscle homogenate was prepared using ice cold (phosphate buffered saline) PBS with Sigmafast, protease inhibitor cocktail (Sigma-Aldrich SA: (animoethyl) benzenesulfonyl fluoride hydrochloride, 2 mM; Aprotinin, 0.3 μM; Bestatin, 130 μM, EDTA, 1 mM E-46 {[(4-guanidinobutyl) amino] -4-methyl-1-oxopentan-2-yl} cyclopropanecarboxylic acid) 14 μM and Leupeptin, 1 μM at pH 7.4 in a ratio of 1:10 (tissue: PBS) using Potter Elvehjem with a glass Teflon homogenizer. The homogenates were taken through three consecutive overnight freeze at − 40 °C (Antech freezer, Qingdao, China) and thaw (room temperature) cycles to break the membrane bilayer and release the embedded proteins as recommended by manufacturer and as described previously (Han et al., [36]). On the fourth day after thawing, the homogenates were centrifuged at 3000 rpm at 4 °C for 15 min and immediately used for GLUT4 determination using Elabscience ELISA kit (catalogue E-EL-R0430) as per manufacturer’s protocol.

### Statistical analysis

Data was analyzed using GraphPad Prism Version 5® software (GraphPad Software Inc., San Diego, CA, USA). Shapiro-Wilks test was used to test for normality and the data was normally distributed. Thus, parametric tests were used for analysis. All results were presented as mean ± standard error of the mean. One-way analysis of variance followed by Tukey’s post hoc test was done to compare differences among multiple groups. Differences between treatment groups was considered significantly different when *p* ≤ 0.05.

## Results

### Phenolic profile by LC-MS analysis

Profiling by LC-MS showed the presence of phenolic phytochemicals including trans-cinnamic acid, syringaldehyde, vanillic acid, protocatechuic acid, syringic acid, p-coumaric acid, gallic acid, ferulic acid and caffeic acid with the latter having the highest relative abundance followed by vanillic and syringic acids. Results are summarised in Table 2.

**Table 2:** Phenolic acids identified in tea samples of *Athrixia phylicoides* herbal tea by LC-MS

Compound	Concentration (μg/L)	Percent (%)
Trans-cinnamic acid	40.22 ± 5.7	3.73
Syringaldehyde	58.08 ± 9.1	5.38
Vanillic acid	186.8 ± 1.8	17.30
Protocatechuic acid	14.91 ± 1.23	1.38
Syringic acid	74.98 ± 1.8	6.95
p-coumaric acid	54.59 ± 2.9	5.06
Gallic acid	10.90 ± 0.8	1.01
Ferulic acid	41.95 ± 1.2	3.89
Caffeic acid	597.1 ± 11.1	55.31

Values expressed as mean ± standard error of mean, *n* = 3

### Effect of treatment with *A. phylicoides* on energy intake, body weight gain and visceral fat accumulation

All treatment groups had similar caloric intake. Despite similar caloric intake, rats treated with both doses of AP had lower net body weight gain (*p* < 0.05) compared to HED fed rats and were similar to ND fed rats. Rats treated with metformin had no effect on net body weight gain which remained similar to both control groups. The HED fed rats had increased visceral fat accumulation (*p* < 0.05) compared to ND fed rats. Treatment with both doses of AP reduced visceral fat accumulation *(p* < 0.05) as compared to HED, although it was not comparable (*p* < 0.05) to ND control. However, metformin had no effect on visceral fat accumulation as it remained similar (*p* >0.05) to HED fed rats (Table 3).

**Table 3:** Effect of AP tea infusion on feed intake, caloric intake, net body weight gain and visceral fat in rats fed with high energy diet

Items	Groups				
	ND control	HED control	HED + AP 50 mg/Kg BW	HED + AP 100 mg/Kg BW	HED + 50 mg/Kg BW metformin
Feed intake, g	268.80 ± 28.80	172.50 ± 15.78^*^	200.00 ± 12.46	197.30 ± 7.44	181.8 ± 15.74^*^
Calorie intake, Kcal/100 g BW	31.48 ± 3.23	32.83 ± 2.93	38.70 ± 2.53	38.66 ± 1.68	39.52 ± 2.97
Net body weight gain, g	29.33 ± 4.06	30.5 ± 2.83	17.33 ± 4.83^#^	19.33 ± 4.19^#^	27.2 ± 3.25
Visceral fat, g	2.02 ± 0.11	4.96 ± 0.35^***^	3.06 ± 0.35^*##^	3.20 ± 0.37^*#^	5.31 ± 0.38^***^
Visceral fat, %	0.50 ± 0.03	1.23 ± 0.10^***^	0.79 ± 0.08^*#^	0.83 ± 0.08^*#^	1.33 ± 0.11^***^

Data expressed as mean ± standard error of mean

*ND*_*C*_ normal diet control, *HED*_*C*_ high energy diet control, *HED*_*L*_ high energy diet + 50 mg/Kg BW AP (*n* = 6), *HED*_*H*_ high energy diet + 100 mg/kg BW AP, *HED*_*M*_ high energy diet + 50 mg/Kg BW metformin (*n* = 6)

*p < 0.05

***p < 0.001 compared to ND_C_

^#^*p* < 0.05

^##^*p* < 0.01 compared to HED_C_

### Effect of treatment with *A. phylicoides* on total blood cholesterol and serum FFAs

**Fig. 1:**
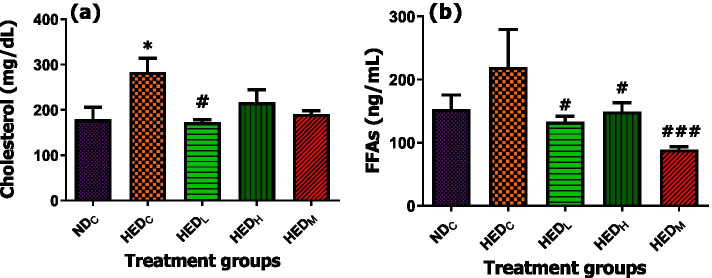
Effect of treatment with AP tea infusion on selected dylipidaemic parameters. **a** Total plasma cholesterol and **b** serum FFAs of HED fed animals. Data expressed as mean ± standard error of mean. ND_C_ = normal diet control; HED_C_ = high energy diet control; HED_L_ = high energy diet + 50 mg/Kg BW AP; HED_H_ = high energy diet + 100 mg/kg BW AP; HED_M_ = high energy diet + 50 mg/Kg BW metformin; FFAs, free fatty acids. *p < 0.05, compared to ND_C_. ^#^*p* < 0.05; ^###^*p* < 0.001 compared to HED_C_

The HED fed rats had increased total blood cholesterol (*p* < 0.05) compared to ND fed rats although FFAs were similar (*p*>0.05) among the control groups and AP treated groups. Only treatment with low dose (50 mg/Kg BW) AP reduced (*p* < 0.05) total blood cholesterol compared to HED fed rats. Both doses of AP and metformin treatment reduced FFAs levels (*p* < 0.05) compared to HED fed rats (Fig. 1).

### Effect of treatment with *A. phylicoides* on adipokine balance

**Fig. 2:**
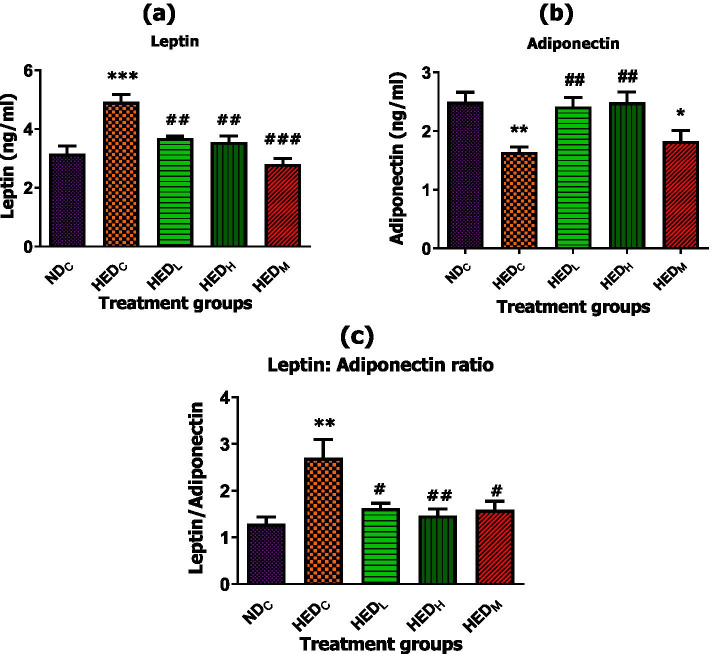
Effect of AP tea infusion on adipokines balance. **a** Serum leptin levels, **b** serum adiponectin levels and **c** leptin: adiponectin levels after 10 week treatment. Data expressed as mean ± standard error of mean. ND_C_ = normal diet control; HED_C_ = high energy diet control; HED_L_ = high energy diet + 50 mg/Kg BW AP; HED_H_ = high energy diet + 100 mg/kg BW AP; HED_M_ = high energy diet + 50 mg/Kg BW metformin. *p < 0.05; **p < 0.01; ***p < 0.001 compared to ND_C_. ^#^*p* < 0.05; ^##^*p* < 0.01; ^###^*p* < 0.001 compared to HED_C_

The HED increased leptin levels, lowered adiponectin levels thereby increased LAR (*p* < 0.05) in HED fed rats compared to ND fed rats. Treatment with both doses of AP tea infusion reduced leptin levels, increased adiponectin levels and lowered LAR (*p* < 0.05) compared to HED fed rats. Metformin similarly reduced (*p*<0.05) leptin levels although adiponectin levels remained similar (*p*>0.05). LAR was reduced (*p* < 0.05) by metformin compared to HED fed rats (Fig. 2).

### Effect of treatment with *A. phylicoides* on blood glucose, insulin, insulin resistance and muscle glucose transporter 4

**Fig. 3:**
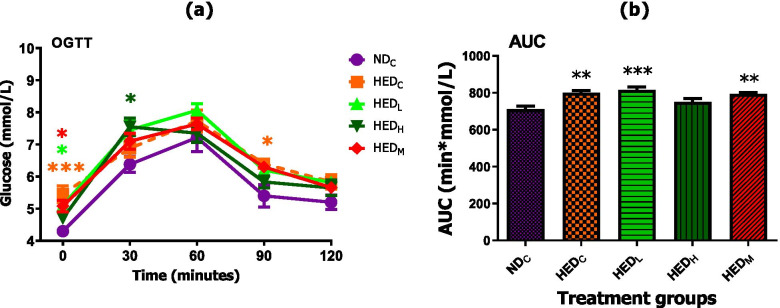
Effect of AP tea infusion on blood glucose. **a** Glucose tolerance and **b** area under the curve (AUC). Data expressed as mean ± standard error of mean. ND_C_ = normal diet control; HED_C_ = high energy diet control; HED_L_ = high energy diet + 50 mg/Kg BW AP; HED_H_ = high energy diet + 100 mg/kg BW AP; HED_M_ = high energy diet + 50 mg/Kg BW metformin. *p < 0.05, **p < 0.01, ***p < 0.001; compared to ND_C_

The HED fed rats including low dose (50 mg/Kg BW) AP and 50 mg/Kg BW metformin treatment groups had increased FBG, sluggish dose response and increased total area under the curve compared to ND fed rats. Only treatment with high dose (100 mg/Kg BW) AP reduced (*p* < 0.05) FBG. High dose (100 mg/Kg BW) AP had a transitional effect as depicted by dose response and total area under the curve similar to ND control group (Fig. 3).

The HED fed rats had increased insulin resistance (*p* < 0.05) compared to ND fed rats. Only treatment with low dose (50 mg/Kg BW) AP tea infusion reduced circulating insulin levels and insulin resistance (*p* < 0.05). Treatment with both doses of AP increased total muscle GLUT4 levels (*p* < 0.05) compared to HED fed rats. Metformin treated rats had lowered (*p* < 0.05) total muscle GLUT4 concentration compared to both control groups (Table 4).

**Table 4:** Effect of AP tea infusion fasting blood glucose, serum insulin, homeostatic assessment of insulin resistance and sensitivity and glucose transporter 4 in rats fed with high energy diet

Items	Groups				
	ND control	HED control	HED + AP 50 mg/Kg BW	HED + AP 100 mg/Kg BW	HED + 50 mg/Kg BW metformin
Fasting	4.30 ± 0.08	5.45 ± 0.27^***^	5.10 ± 0.13^*^	4.70 ± 0.09^#^	5.08 ± 0.18^*^
Insulin	0.80 ± 0.23	0.67 ± 0.17	0.28 ± 0.07^#^	0.76 ± 0.09	0.63 ± 0.30
HOMA-IR	1.06 ± 0.40	2.94 ± 0.53^*^	1.00 ± 0.21^#^	2.35 ± 0.28	1.12 ± 0.41
GLUT4	8.21 ± 0.51	7.60 ± 0.12	9.78 ± 1.01^#^	9.07 ± 0.34^###^	6.60 ± 0.55^*#^

Data expressed as mean ± standard error of mean

*ND*_*C*_ normal diet control, *HED*_*C*_ high energy diet control, *HED*_*L*_ high energy diet + 50 mg/Kg BW AP, *HED*_*H*_ high energy diet + 100 mg/kg BW AP, *HED*_*M*_ high energy diet + 50 mg/Kg BW metformin

*p < 0.05

**p < 0.01

***p < 0.001; compared to ND_C_

^#^*p* < 0.05

^###^*p* < 0.001 compared to HED

## Discussion

The Diet-induced MetS based on high-carbohydrate dietary intervention represents a valuable tool to investigate and validate new therapeutic possibilities in the treatment of obesity, diabetes and other cardiovascular risk factors. Without any doubt, our study confirmed components of diet-induced MetS, namely body weight gain, visceral adiposity, FFA, adipokine dysregulation (i.e. inflammation), hyperglycemia, insulin resistance, and hypercholesterolemia.

Natural teas rich in polyphenols with beneficial effects on metabolic derangements have gained interest by researchers [22, 37]. These natural teas may offer a multi-target effect with lesser side effects on obesity related derangements and are easily accessible by poor communities residing in villages. Several human studies have proved that tea drinking could alleviate MetS [25, 26]. Our findings confirmed AP to be rich in polyphenols, especially caffeic, vanillic and syringic acids as has been previously reported [24]. Considering that polyphenols in AP including flavonoids and tannins have been reported to be responsible for its medicinal properties [24, 25], in this present study, we assessed the effect of AP on diet-induced MetS in rats.

Obesity which is central to the development of MetS and commonly results from the consumption of HED diets is known to promote the accumulation of fats in the adipose tissue and increase circulatory cholesterol in the body [3]. Excess fat storage in the adipose tissue as TG leads to adipocyte hypertrophy which promotes lipolysis leading to the release of FFAs into the circulation [38]. As confirmed by our study, HED fed rats had increased visceral fat, total cholesterol and FFAs which were reduced by AP treatment. Increased visceral fat accumulation and adipocyte hypertrophy is associated with adipokine dysregulation, which includes increased pro-inflammatory leptin and reduced anti-inflammatory adiponectin concentrations in the circulation [17]. Traditionally, leptin is known to increase appetite, body weight and thermogenesis, and reduce energy expenditure [39]. Obesity is associated with overproduction of leptin, leading to leptin-induced local and peripheral inflammation [40] via the activation of TNF-α and IL-6 production, and stimulation of surface markers [41]. In our study, HED fed rats had increased leptin concentrations which were reduced by treatment with AP tea infusion as well as metformin. Hamdaoui et al. reported similar results where black and green tea infusions decreased leptin levels in obese rats [42].

Adiponectin is another adipocyte-derived cytokine that is known to possess anti-MetS effects through anti-obesity and anti-diabetic actions and alleviates insulin resistance by stimulation of lipid breakdown and inhibiting inflammatory responses and atherosclerosis [43]. HED has been demonstrated to decrease adiponectin concentration, which is consistent with the current study [44]. However in this study, hypoadiponectinaemia was ameliorated by treatment with AP tea infusion. Similar results were also reported in other studies that used medicinal plants such as green tea in rat models [44] and oolong tea in humans [45]. Tian et al. reported that green tea increased adiponectin concentration by reversal of reduced peroxisome proliferator-activated receptor (PPARγ) in HED fed rats [44]. PPARγ binds with PPAR-responsive element to stimulate transcription of the adiponectin gene, thereby increasing adiponectin concentration [46]. It is conceivable that AP tea infusion may follow the same mechanism of action to increase adiponectin concentrations in HED fed rats. Studies have reported that increased LAR is strongly associated with MetS and cardiovascular diseases than isolated leptin or isolated adiponectin concentrations [16, 17]. López-Jaramillo et al. further suggested that the LAR may be a useful diagnostic index for insulin resistance and a good marker for assessing the effectiveness of antidiabetic therapy [16]. In this study, treatment with AP reduced LAR in HED animals. This finding suggest that AP possess anti-inflammatory properties. A previous study has equally shown Catechin-rich green tea extract to ameliorate mucosal inflammation in mice [47].

Obesity-induced inflammation is closely associated with IR in adipocytes, hepatocyte and other insulin-sensitive tissues through several pathways. Insulin plays an important role in glycaemic control, and deficient insulin actions may cause glucose intolerance and may progress to type 2 diabetes mellitus [48]. Our study showed that AP improved hyperglycaemia and the effect was dose dependent as the higher dose of AP (100 mg/Kg BW + AP) reduced FBG and postprandial blood glucose (PBG) following an OGTT through week 8. It is suggested that a decrease in glucose clearance from the circulation occurs with impaired insulin secretion and sensitivity [47]. Our data showed that the low dose AP tea infusion improved glucose tolerance, and reduced circulating insulin and IR. Similar results were reported for green tea, where hyperinsulinaemia and IR were ameliorated in mice [49]. Amelioration of hyperinsulinaemia has been attributed to insulin-degrading enzyme [50]. Therefore, it may be suggested that AP can reduce IR by promoting insulin clearance through the inhibition of insulin degrading enzyme activity as reported for green tea [49]. It is well known that long term glycaemic control in the body primarily depends on appropriate insulin secretion from pancreatic beta cells and tissue sensitivity toward insulin to increase glucose uptake by specific transporters such as GLUT4 [51]. Our findings showed AP tea infusion to increase GLUT4 in the muscle of animals suggesting it promotes the transportation of glucose into the cell for metabolism. *Athrixia phylicoides* have shown to have some direct effects on the improvement of glycaemic control as demonstrated by improved FBG, insulin action and increase GLUT 4 transporter for glucose metabolism. Various teas have been shown to interfere with carbohydrate digestion, absorption and metabolism through their abilities to inhibit α-amylase [52], α-glucosidase 4 [53], intestinal glycosidase activity [54] and promote the expression [55] and translocation [56] of GLUT4 transporter. The potential mechanism of AP tea anti-diabetic activity may be through the enhancement of insulin sensitivity which induces the expression and translocation of GLUT4 transporter in glucose metabolic cells. The activation of GLUT4 facilitates glucose update and metabolism thereby enhancing glucose tolerance and lowering blood glucose levels.

Adiposity and associated IR play a critical role in the development of dyslipidaemia. Adipose tissue IR is associated with increased lipolysis [57]. The inhibitory effect of insulin on lipolytic hormone-sensitive lipase in adipose tissues and stimulation of lipogenic lipoprotein lipase in peripheral tissues is reduced in the state of IR resulting in overall lipolysis [58]. Therefore, increased lipolysis within the adipose tissue results in high FFA flux into the circulation which may affect insulin signaling pathway leading to IR [59]. Also, increase in visceral fat is associated with inflammation, inversely proportional to insulin secretion and sensitivity, and directly proportional to IR [60]. The effect of AP tea infusion on glycaemic control in our study may be partially attributable to the observed decrease in weight gain, reduced visceral fat accumulation, reduced FFAs and reduced LAR which all have an indirect effect in ameliorating IR.

Metformin is a known drug used to manage diabetes with several anti-diabetic effects. Apart from its hyperglycaemic effect and improvement of IR, metformin is known to inhibit and reduce visceral fat accumulation in humans [61] and mice [62]. It has been reported to inhibit the mitogen-activated protein kinase (MAPK) signaling pathway in adipocytes leading to reduction of leptin [63]. It has been reported to ameliorate hypoadiponectinaemia in type 2 diabetic rats [64]. In this study, AP teas infusion was shown to have similar anti-diabetic effects as metformin in ameliorating IR and lowering blood glucose as well as upregulate adiponectin and reducing leptin and FFAs. This finding suggest that AP tea may exert its anti-diabetic effects through similar mechanism as metformin. More so, AP showed a better effect in lowering weight gain, visceral fat and total cholesterol, and upregulating adiponectin than metformin. Therefore, apart from its anti-diabetic effect, AP tea may have added advantage over metformin with its anti-obesity effects and has potential to be used as a remedy for weight loss. Although this study has shown AP tea extract to improve some parameters of MetS, this study did not directly assess triglyceride and HDL-c which are key components of dyslipidaemia and MetS. However, this study assessed visceral fat, FFA and cholesterol which are markers of adiposity and dyslipdaemia implicated in MetS. More so, only male animals were used in the study because female animals became pregnant which could affect the outcome of the study. Therefore, it will be of interest in future to assess the effect of AP on all component of the MetS including key biomarkers of the metabolic pathway using both male and female animals to enhance the findings of this study and better elucidate the mechanism of action of AP on MetS.

## Conclusion

This study showed *Athrixia phylicoides* tea infusion to reduce central adiposity, restored adipokine regulation, ameliorated hyperglycemia, improve glucose uptake and reduce insulin resistance in HED diet-induced MetS in rats. The presence of polyphenols in *Athrixia phylicoides* tea may be responsible for the observed biological effects linked to its medicinal properties. *Athrixia phylicoides* tea infusion have potential to be used as a remedy for type 2 diabetes and obesity which are components of MetS. Our study has added a new understanding on how natural products such as AP tea infusion affects adipokine balance, lipid and glucose homeostasis in vivo.

## Data Availability

All data generated or analysed during this study are included in this published article.

## Abbreviations

MetS: Metabolic syndrome
HED: High fat/energy diet
FFA: Free fatty acid
TG: Triglyceride
LDL-c: Low density lipoprotein cholesterol
HDL-c: High density lipoprotein cholesterol
TNF-α: Tumour necrosis factor-alpha
IL: Interleukin
LAR: Leptin to adiponectin ratio
IR: Insulin resistance
AP: *Athrixia phylicoides*
LC-MS: Liquid chromatography- mass spectrometer
QTOF: Quadrupole time-of-flight
MS: Mass spectrometer
UPLC: Ultra-performance liquid chromatography
EDTA: Ethylenediaminetetraacetic acid
GLUT 4: Glucose transporter 4
ELISA: Enzyme linked immunosorbent assay
HOMA-IR: Homeostasis model assessment of insulin resistance
PPARγ: Peroxisome proliferator-activated receptor
OGTT: Oral glucose tolerance test
FBG: Fasting blood glucose
PBG: Postprandial blood glucose
MAPK: Mitogen-activated protein kinase
